# Effect of photoperiod on the feline adipose transcriptome as assessed by RNA sequencing

**DOI:** 10.1186/1746-6148-10-146

**Published:** 2014-07-03

**Authors:** Akihiro Mori, Kelly L Kappen, Anna C Dilger, Kelly S Swanson

**Affiliations:** 1 Department of Animal Sciences, University of Illinois, 1207 West Gregory Drive, 162 Animal Sciences Laboratory, Urbana, IL 61801, USA

**Keywords:** Cat, Gene expression, Adipose tissue, RNA-seq, Transcriptomics

## Abstract

**Background:**

Photoperiod is known to cause physiological changes in seasonal mammals, including changes in body weight, physical activity, reproductive status, and adipose tissue gene expression in several species. The objective of this study was to determine the effects of day length on the adipose transcriptome of cats as assessed by RNA sequencing. Ten healthy adult neutered male domestic shorthair cats were used in a randomized crossover design study. During two 12-wk periods, cats were exposed to either short days (8 hr light:16 hr dark) or long days (16 hr light:8 hr dark). Cats were fed a commercial diet to maintain baseline body weight to avoid weight-related bias. Subcutaneous adipose biopsies were collected at wk 12 of each period for RNA isolation and sequencing.

**Results:**

A total of 578 million sequences (28.9 million/sample) were generated by Illumina sequencing. A total of 170 mRNA transcripts were differentially expressed between short day- and long day-housed cats. 89 annotated transcripts were up-regulated by short days, while 24 annotated transcripts were down-regulated by short days. Another 57 un-annotated transcripts were also different between groups. Adipose tissue of short day-housed cats had greater expression of genes involved with cell growth and differentiation (e.g., myostatin; frizzled-related protein), cell development and structure (e.g., cytokeratins), and protein processing and ubiquitination (e.g., kelch-like proteins). In contrast, short day-housed cats had decreased expression of genes involved with immune function (e.g., plasminogen activator inhibitor 1; chemokine (C-C motif) ligand 2; C-C motif chemokine 5; T-cell activators), and altered expression of genes associated with carbohydrate and lipid metabolism.

**Conclusions:**

Collectively, these gene expression changes suggest that short day housing may promote adipogenesis, minimize inflammation and oxidative stress, and alter nutrient metabolism in feline adipose tissue, even when fed to maintain body weight. Although this study has highlighted molecular mechanisms contributing to the seasonal metabolic changes observed in cats, future research that specifically targets and studies these biological pathways, and the physiological outcomes that are affected by them, is justified.

## Background

Photoperiod is known to alter many physiologic outcomes in seasonal animals, including food intake, weight gain, and estrous. Melatonin, secreted by the pineal gland, is an important signal contributing to these seasonal responses. Melatonin secretion exhibits an obvious circadian rhythm, peaking during the dark period [1]. Over the course of a year, the peak of melatonin concentrations and the duration of high melatonin production is greatest during winter/short days (SD) and lowest during summer/long days (LD) [2]. The response to decreased daylight depends on species, however, with some gaining weight and others losing weight. For example, Siberian hamsters and meadow voles lose fat mass and total body weight (BW) with shortened days [3,4], while Syrian hamsters, collared lemmings, prairie voles and raccoon dogs gain fat mass and total BW with SD [5-8]. Cats are sensitive to photoperiod as it relates to circulating melatonin concentrations and estrous cycling [9-11] and seasonal differences in activity levels are known to exist in feral cat populations, with increased activity during the spring/summer period [12]. Seasonal and photoperiod changes on energy intake, energy expenditure, and voluntary physical activity levels have also been recently reported in domestic cats [13,14]. Feline obesity continues to increase in the pet population and is a risk factor for many chronic diseases. Although studying seasonal changes may provide a better understanding of energy homeostasis in cats, the mechanisms by which these changes occur in adipose tissue have not been adequately studied.

Studies in ruminants and rodents have shown that seasonal BW changes are often due to seasonal cycles in fat deposition and adipose tissue lipogenic activity and gene expression [4,15-17]. Leptin, an important long-term BW regulator, is a common molecular target of study. Although leptin is typically positively correlated with BW, the mRNA expression and secretion of leptin also undergoes significant seasonal fluctuations. Adipose tissue leptin mRNA has been shown to increase during LD in several species, including lactating dairy cows, ovariectomized ewes, and Siberian hamsters [16,18-20]. These increases did not necessarily correlate with BW, however, suggesting that photoperiod may override leptin responsiveness in some situations. A great example of this response has been reported in Siberian hamsters housed in LD vs. SD. In that study, food intake was altered due to photoperiod, but did not correlate with adipose tissue leptin mRNA abundance [18]. Thus, it appears that leptin sensitivity and signaling in Siberian hamsters is dependent on day length [18], with increased leptin resistance during LD [21]. Enzymes involved in adipose tissue metabolism have also been studied in several animal models, but with conflicting results [20,22-24].

While it is clear that photoperiod impacts BW and adipose tissue metabolism, the response appears to be species-specific. Although previous studies have identified mechanisms by which ovariohysterectomy and/or dietary intervention contribute to changes in adipose tissue metabolism and BW in cats [25-27], a molecular analysis of how photoperiod affects the adipose tissue transcriptome has not yet been performed in this species. Moreover, only a few select genes or biological pathways have been typically studied in this regard. Therefore, research performed specifically in the cat using high-throughput molecular techniques to evaluate the entire adipose tissue transcriptome was justified. A commercial feline microarray is not currently available so RNA sequencing (RNA-seq), which provides a global assessment of gene expression [28,29], was deemed to be the best strategy to use in this study. Thus, our objective was to evaluate the effects of photoperiod on the adipose transcriptome in cats as measured by RNA-seq. To test the effects of photoperiod only, and not the change in BW that often occurs with altered day length, cats were fed to maintain BW throughout the study.

## Methods

### Ethics statement

All animal procedures were approved by the University of Illinois Institutional Animal Care and Use Committee prior to animal experimentation.

### Animals and treatments

Ten healthy adult (4 yr old) neutered male domestic shorthair cats of healthy body condition (5/9 body condition score) were used. Cats were group housed for 22 hr of each day, but were isolated in individual stainless steel cages (0.61 × 0.61 × 0.61 m) for 1 hr twice daily (9:00–10:00 am; 4:00–5:00 pm) so food could be offered and individual intake measured. One group of cats (n = 5) was housed in a room measuring 12 m^2^ and a second group of cats (n = 5) was housed in a room measuring 26 m^2^. Cats had access to various toys and scratching posts for behavioral enrichment. Cats were fed a dry commercial diet (Whiskas® Meaty Selections®, Mars, Inc., Mattoon, IL) to maintain BW (within 5% of baseline BW) throughout the experiment. Water was available *ad libitum* throughout the experiment.

Four weeks prior to the experiment (wk -4), cats were acclimated to the diet and daily feeding schedule, and were exposed to 12 hr light: 12 hr dark. The experiment was performed using a crossover design composed of two 12-wk periods. Cats were randomly assigned to one of two groups, and with groups being randomly assigned to room. Cats remained in the same rooms throughout the experiment. Rooms were adjacent to each other in the animal facility, were maintained at the same temperature (20°C to 22°C) and humidity level (30% to 70%) throughout the study, and did not have windows (a source of natural light). For the first 12-wk period, Group 1 was exposed to 16 hr light: 8 hr dark (LD), while Group 2 was exposed to 8 hr light: 16 hr dark (SD). For the second 12-wk period, Group 1 was exposed to SD and Group 2 was exposed to LD. Daily food intake and twice-weekly BW were measured throughout the study. Food intake was adjusted to maintain BW and body condition score (9 point scale) [30] throughout the study.

### Body composition, physical activity, and indirect calorimetry

Body composition, voluntary physical activity, and indirect calorimetry were measured during wk 0 and 12 as described previously [14]. Briefly, body composition (lean mass, fat mass, and bone mineral mass) was measured by dual-energy X-ray absorptiometry. Cats were placed in ventral recumbency and body composition was analyzed using a Hologic model QDR-4500 Fan Beam X-ray Bone Densitometer and software (Hologic Inc., Waltham, MA). Voluntary physical activity was measured using Actical activity monitors (Mini Mitter, Bend, OR). Activity collars were worn around the neck for 7 consecutive d. Collars were then removed and data were analyzed, compiled, and converted into arbitrary numbers referred to as ‘activity counts’ by Actical software. Activity counts were summed and recorded at intervals of 15 sec. Activity data are represented as activity counts per hr. Indirect calorimetry was measured in calorimetry chambers measuring 0.52 × 0.52 × 0.41 m. Respiratory exchange ratio, heat, and flow of air through the chambers were measured using an open-circuit Oxymax System (Software, Version 5.0, Columbus Instruments, Columbus, OH). Cats were food restricted overnight before indirect calorimetry and acclimated to the calorimetry chambers for at least 4 hr, after which time measurements were collected for 2 hr. Data from these 2 hr were extrapolated to obtain 24 hr daily energy expenditure values.

### Adipose tissue sample collection and handling

Adipose tissue biopsies were collected at wk 12 of each period for RNA isolation and sequencing. After withholding food for at least 12 hr, cats were sedated with butorphanol (0.2 mg/kg), atropine (0.04 mg/kg) and medetomadine (0.02 mg/kg; intramuscular). Approximately 1 g of subcutaneous abdominal adipose tissue was collected, immediately flash frozen in liquid nitrogen, and stored at -80°C until further analyses.

### RNA extraction

Total cellular RNA was isolated from adipose samples using the Trizol reagent as suggested by the manufacturer (Invitrogen, Carlsbad, CA). RNA concentration was determined using a ND-1000 spectrophotometer (NanoDrop Technologies, Wilmington, DE, USA). RNA integrity was confirmed using a 1.2% denaturing agarose gel.

### Construction of RNA-seq libraries

Construction of libraries and sequencing on the Illumina HiSeq2000 was performed at the W. M. Keck Center for Comparative and Functional Genomics at the University of Illinois at Urbana-Champaign (Urbana, IL). RNA-seq libraries were constructed using the TruSeq RNA Sample Preparation Kit (Illumina San Diego, CA). Briefly, mRNA was selected from one microgram of high quality total RNA. First-strand synthesis was synthesized with a random hexamer and SuperScript II (Life Technologies Corp., Carlsbad, CA). Double-stranded DNA was blunt-ended, 3′-end A-tailed and ligated to indexed adaptors. The adaptor-ligated double-stranded cDNA was amplified by PCR for 10 cycles with the Kapa HiFi polymerase (Kapa Biosystems, Woburn, MA) to reduce the likeliness of multiple identical reads due to preferential amplification. The final libraries were quantified using a Qubit (Life Technologies, Grand Island, NY) and the average size was determined on an Agilent bioanalyzer DNA7500 DNAchip (Agilent Technologies, Wilmington, DE), diluted to 10 nM and the indexed libraries were pooled in equimolar concentrations. The pooled libraries were further quantified by qPCR on an ABI 7900, which resulted in high accuracy and a maximization of cluster numbers in the flowcell.

### Illumina sequencing

The multiplexed libraries were loaded onto three lanes of an 8-lane flowcell for cluster formation and sequenced on an Illumina HiSeq2000. One of the lanes was loaded with a PhiX Control library that provides a balanced genome for calculation of matrix, phasing and pre-phasing, which are essential for accurate base calling. The libraries were sequenced from one end of the molecules to a total read length of 100 nt. The raw .bcl files were converted into de-multiplexed fastq files with Casava 1.8.2 (Illumina, CA).

### RNA-sequence alignment and statistical analysis

Raw FASTQ data were quality-trimmed from the 5′ and 3′ ends using the program Seqtk (http://github.com/lh3/seqtk), using a minimal PHRED quality score of 20 and a minimal length of 25. Sequences were then aligned using TopHat v. 2.0.6 [31] and Bowtie 2.0.4 [32]. The reference genome annotation and sequence index were from Felis_catus_6.2 from Ensembl (http://useast.ensembl.org/Felis_catus/Info/Annotation/#assembly). We used the GTF gene model file provided by The Sanger Centre for splice junctions and read counts (ftp://ftp.sanger.ac.uk/pub/users/searle/cat). Each file was sorted using novosort (http://www.novocraft.com/wiki/tiki-index.php?page=Novosort&structure=Novocraft+Technologies&page_ref_id=91) prior to read counting. Raw read counts were tabulated from each sample from the generated BAM alignments at the gene level using htseq-count, from HTSeq v0.5.4p1 (http://www-huber.embl.de/users/anders/HTSeq/doc/index.html).

The raw read counts were input into R 2.15.1 [33] for data pre-processing and statistical analysis using packages from Bioconductor [34] as indicated below. Genes without 1 count per million mapped reads in at least one of the samples were filtered out due to unreliable data in any sample. Those genes that passed this filter were analyzed using edgeR 2.6.10 [35]. The raw count values were used in a negative bionomial statistical model that accounts for the total library size for each sample and an extra normalization factor [36] for any biases due to changes in highly expressed genes [37]. For sample clustering and heatmaps, comparable expression values were generated from the read counts using the voom transformation from limma 3.12.1 [38]. Gene symbols, descriptions, and Gene Ontology terms for the Ensembl gene models were downloaded from Ensembl Genes 70, Felis_catus_6.2. All raw sequence data were deposited in the Gene Expression Omnibus repository at the National Center for Biotechnology Information (NCBI) archives (http://www.ncbi.nlm.nih.gov/geo) under accession #GSE46431 (GSM1130067-GSM1130086).

## Results and discussion

Obesity is a common health problem in the domestic cat population. Common therapeutic options include dietary intervention, mainly by energy restriction, and increased physical activity. Although these strategies are logical, in practice, they are not often successful. A better understanding of the molecular changes that occur in metabolically active tissues, including adipose tissue, may increase our understanding of feline metabolism, identifying methods for promoting weight maintenance. Photoperiod has been shown to alter the metabolism and BW of many seasonal animals, but has not been well studied in cats. Because their breeding habits and physical activity levels are altered by season [12-14], we believe that studying photoperiod may identify novel strategies to improve weight maintenance in pet cats. In this study, our objective was to use RNA-seq to identify genes and biological pathways differentially expressed in adipose tissue of lean adult cats housed in SD vs. LD conditions. So that we could study the effects of photoperiod using a crossover design and without the bias of BW change between periods, cats were fed to maintain BW throughout the entire study. Although the effects of photoperiod in our study may differ from cats fed *ad libitum*, we believed this study design was most appropriate to identify photoperiod-induced changes without the bias of BW gain.

As described above and presented by Kappen et al. [14], cats were fed to maintain BW (4.6 kg) and body condition score throughout the study. Cats were composed of approximately 16% fat mass, 82% lean mass, and 2% bone mass, values that were not altered by photoperiod. To maintain BW, however, SD-housed cats required less (P < 0.0001) energy than LD-housed cats (187 vs. 196 kcal/d). Similarly, SD-housed cats had reduced (P = 0.008) physical activity as assessed by accelerometers (3129 vs. 3770 activity counts/hr) and reduced (P < 0.05) resting metabolic rate as assessed by indirect calorimetry (8.4 vs. 9.0 kcal/hr) [14]. Because photoperiodic history may impact response to day length, the history of the cats studied herein is important. For most of their lives, the cats used in this study have been housed in an indoor animal facility without exposure to natural light and under a 16 hr light: 8 hr dark photoperiod. This light cycle has been used in our facility for many years due to practical reasons. It allows us to perform a variety of study designs and allows the collection of samples (blood, fecal, or urine), feeding, weighing, etc. early in the morning or into the evening when the lights are on in the facility (6 am to 10 pm). Because the current study was performed as a crossover design and 12-wk periods were used, previous history likely did not impact the gene expression results presented here, but it cannot be ruled out completely.

Because commercial DNA microarrays are not currently available for cats, we used RNA-seq to analyze global gene expression profiles of adipose tissue in this study. A total of 578 million sequences (28.9 million/sample) were generated by Illumina sequencing. Using a raw p value of P < 0.005, 170 mRNA transcripts were differentially expressed between SD- and LD-housed cats. Of the 170 transcripts highlighted, 89 annotated transcripts were up-regulated by SD, while 24 annotated transcripts were down-regulated by SD. Another 57 un-annotated transcripts (name and function not known) were also different between groups. Annotated, non-redundant genes were then classified into functional classes using SOURCE (http://smd.princeton.edu) [39] and NCBI resources (http://www.ncbi.nlm.nih.gov).

**Figure 1:**
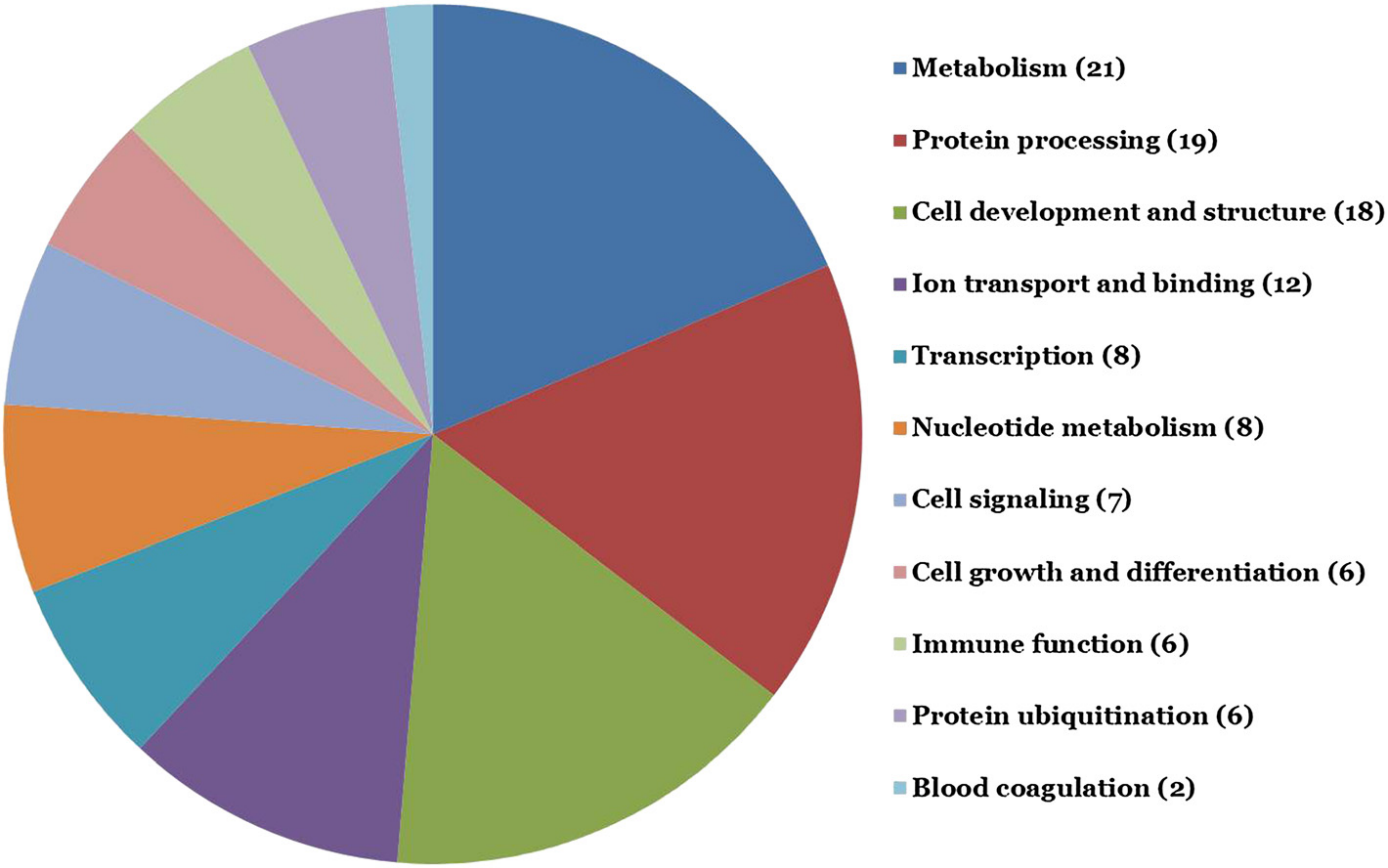
Major functional gene classes affected by photoperiod in feline adipose tissue.

Adipose tissue is complex and contains many different cell types, including adipocytes, preadipocytes, immune cells, endothelial cells, and fibroblasts. Our data are derived from adipose tissue biopsies, so gene expression differences cannot be attributed to different cellular fractions or types, but represents changes in gene expression of whole adipose tissue. Nonetheless, according to gene ontology classification, expression of genes in several broad functional categories, including protein processing and ubiquitination, nucleotide metabolism, transcription, cell growth and differentiation, cell development and structure, cell signaling, immune function, metabolism, and ion transport and binding, were altered by photoperiod (Figure 1). Altogether, these gene expression changes suggest that SD housing may promote adipogenesis, minimize inflammation and oxidative stress, and alter lipid and carbohydrate metabolism in adipose tissue of cats. Rather than feeding cats to maintain BW, as was done in this study, *ad libitum* access to food and the allowance of weight gain would probably be needed to test this physiologic response effectively.

Based on the gene expression changes observed in this study, namely the up-regulation of myostatin (MSTN), frizzled-related protein (FRZB), several kelch-like proteins (KLHL23, 33, 34, and 38; KBTBD12), and several genes involved with protein ubiquitination, adipogenesis and adipocyte differentiation appeared to be more abundantly expressed in SD- vs. LD-housed cats (Tables 1 and 2). Melatonin has been shown to promote differentiation of 3T3-L1 fibroblasts *in vitro*[40], but its effects on adipose metabolism of cats *in vivo* have not been described. Adipogenesis is the process by which mesenchymal stem cells differentiate into adipocytes. One of the most important regulators of this process is the Wnt/β-catenin signaling pathway. Constitutive activation of the Wnt/β-catenin pathway in preadipocytes inhibits differentiation by preventing the induction of C/EBPα and PPARγ [41,42]. Conversely, inactivation of this pathway releases the brake on adipogenesis [43-45]. Therefore, increased expression of FRZB, which is known to be a Wnt antagonist [46,47], suggested increased adipogenesis in SD-housed cats. Because cats were not allowed to gain BW or body composition in this study and because excess adipose tissue was not available to measure markers of adipogenesis (e.g., Pref-1 only expressed in pre-adipocytes; FABP4 only expressed in mature adipocytes), it is unknown whether the process was actually up-regulated. MSTN, the expression of which was also increased in SD-housed cats, is a member of the TGF-β superfamily and is most commonly known as an important regulator of skeletal muscle development. While high levels of MSTN are detected in skeletal muscle, low levels are also detected in other tissues, including adipose tissue [48]. Although mixed results have been demonstrated *in vitro*[49,50], MSTN overexpression in adipose tissue *in vivo* results in an increased number of small immature adipocytes, increased energy expenditure, and resistance to obesity [51]. MSTN appears to regulate preadipocyte differentiation and lipid metabolism in adipose tissue via ERK1/2 [52].

**Table 1:** Genes associated with protein processing, nucleotide metabolism, and transcription that had increased expression in adipose tissue of SD- versus LD-housed cats

**Functional classification**	**Gene name**	**Gene symbol**	**Fold change**
Protein processing			
Protein processing	membrane protein, palmitoylated 6 (MAGUK p55 subfamily member 6)	MPP6	1.79
Protein processing	A kinase (PRKA) anchor protein 6	AKAP6	2.99
Protein processing	cadherin 15, type 1, M-cadherin (myotubule)	CDH15	2.46
Protein processing	DnaJ (Hsp40) homolog, subfamily C, member 12	DNAJC12	3.25
Protein processing	karyopherin alpha 3 (importin alpha 4)	KPNA3	2.19
Protein processing	kelch-like 23 (Drosophila)	KLHL23	2.31
Protein processing	kelch-like 33 (Drosophila)	KLHL33	4.31
Protein processing	kelch-like 34 (Drosophila)	KLHL34	3.34
Protein processing	kelch-like 38 (Drosophila)	KLHL38	2.57
Protein processing	kelch repeat and BTB (POZ) domain containing 12	KBTBD12	2.57
Protein processing	myotubularin related protein 7	MTMR7	2.12
Protein processing	membrane protein, palmitoylated 7 (MAGUK p55 subfamily member 7)	MPP7	2.45
Protein processing	myosin light chain kinase family, member 4	MYLK4	4.05
Protein processing	protein kinase C, theta	PRKCQ	3.48
Protein processing	SRSF protein kinase 3	SRPK3	2.81
Protein processing	synemin, intermediate filament protein	SYNM	3.37
Protein processing	triadin	TRDN	4.41
Protein ubiquitination	LIM domain 7	LMO7	3.73
Protein ubiquitination	tripartite motif containing 55	TRIM55	5.17
Protein ubiquitination	ubiquitin protein ligase E3 component n-recognin 3 (putative)	UBR3	1.87
Protein ubiquitination	WW domain containing E3 ubiquitin protein ligase 1	WWP1	2.00
Ubiquitin cycle	cullin 3	CUL3	1.70
Ubiquitin cycle	ubiquitin specific peptidase 28	USP28	2.90
Nucleotide metabolism			
Nucleotide binding	cAMP-regulated phosphoprotein, 21 kDa	ARPP21	3.28
Nucleotide binding	DnaJ (Hsp40) homolog, subfamily C, member 21	DNAJC21	1.93
Nucleotide binding	RNA binding motif protein 24	RBM24	3.50
Nucleotide binding	Ras-related GTP binding D	RRAGD	3.02
Nucleotide binding	zinc finger CCHC-type and RNA binding motif 1	ZCRB1	1.53
Nucleotide metabolism	adenosine monophosphate deaminase 1	AMPD1	4.31
Nucleotide metabolism	nudix (nucleoside diphosphate linked moiety X)-type motif 4	NUDT4	1.85
Nucleotide metabolism	5′-nucleotidase, cytosolic IA	NT5C1A	3.22
Transcription			
Transcription	eyes absent homolog 1 (Drosophila)	EYA1	3.62
Transcription	eyes absent homolog 4 (Drosophila)	EYA4	4.10
Transcription	DDB1 and CUL4 associated factor 6	DCAF6	2.34
Transcription	homeobox A10	HOXA10	2.89
Transcription	MTERF domain containing 3	MTERFD3	1.67
Transcription	muscle-related coiled-coil protein	MURC	5.53
Transcription	RNA binding protein, fox-1 homolog (C. elegans) 1	RBFOX1	3.99

**Table 2:** Genes associated with cell development and structure, cell growth and differentiation, and cell signaling that had increased expression in adipose tissue of SD- versus LD-housed cats

**Functional classification**	**Gene name**	**Gene symbol**	**Fold change**
Cell development and structure			
Actin binding	formin homology 2 domain containing 3	FHOD3	3.99
Actin binding	myosin, heavy chain 3, skeletal muscle, embryonic	MYH3	4.23
Actin binding	myosin, heavy chain 8, skeletal muscle, perinatal	MYH8	4.78
Cell development	homeobox A9	HOXA9	2.18
Cell development	microtubule-associated protein tau	MAPT	3.21
Cell development	myotilin	MYOT	5.42
Cell development and structure	myozenin 3	MYOZ3	4.26
Cell development and structure	xin actin-binding repeat containing 2	XIRP2	5.87
Keratin	keratin 5	KRT5	29.56
Keratin	keratin 14	KRT14	125.24
Keratin	keratin 25	KRT25	7.52
Keratin	keratin 74	KRT74	9.79
Keratin	keratin 85	KRT85	15.94
Keratin associated protein	keratin associated protein 8-1	KRTAP8-1	37.71
Keratin associated protein	keratin associated protein 11-1	KRTAP11-1	53.79
Muscle heart development	calsequestrin 2 (cardiac muscle)	CASQ2	4.06
Skeletal development	C-type lectin domain family 3, member A	CLEC3A	4.14
Cell growth and differentiation			
Cell differentiation	frizzled-related protein	FRZB	4.92
Cell differentiation	leucine-rich, glioma inactivated 1	LGI1	1.87
Cell differentiation	myeloid leukemia factor 1	MLF1	2.88
Cell growth and differentiation	Myostatin	MSTN	7.33
Cell signaling			
Cell signaling	ankyrin 1, erythrocytic	ANK1	3.79
Cell signaling	ankyrin 3, node of Ranvier (ankyrin G)	ANK3	1.93
Cell signaling	ankyrin repeat and SOCS box containing 4	ASB4	3.78
Cell signaling	ankyrin repeat and SOCS box containing 14	ASB14	3.36
Cell signaling	ankyrin repeat and SOCS box containing 15	ASB15	4.54
Cell signaling	CAP, adenylate cyclase-associated protein, 2 (yeast)	CAP2	3.02
Cell signaling	signal transducing adaptor family member 1	STAP1	2.56

LIM domain 7 (LMO7), tripartite motif containing 55 (TRIM55), ubiquitin protein ligase E3 component n-recognin 3 (UBR3), WW domain containing E3 ubiquitin protein ligase 1 (WWP1), cullin 3 (CUL3), and ubiquitin specific peptidase 28 (USP28), all of which are involved in protein ubiquitination and the ubiquitin cycle, were up-regulated in SD-housed cats (Table 1). The ubiquitin–proteasome pathway has a well-established role in a number of physiological and pathological processes and is known to be important in cellular differentiation [53-55]. Proteasome activity has been shown to be at its highest level during the early stages of differentiation in human adipose-derived stem cells [56] and reduced as stem cells become differentiated. KLHL23, KLHL33, KLHL34, KLHL38, and kelch repeat and BTB (POZ) domain containing 12 (KBTBD12) were up-regulated in SD-housed cats (Table 1). Kelch family proteins contain two conserved domains, a BTB domain, and a kelch repeat domain. The kelch repeat domain is involved in organization of the cytoskeleton via interaction with actin and intermediate filaments, whereas the BTB domain has multiple cellular roles, including recruitment to E3 ubiquitin ligase complexes [57,58]. Kelch-related actin-binding protein has previously been shown to be expressed in adipose tissue, specifically in the adipose-derived stroma-vascular fraction [59], and is transiently induced early during adipocyte differentiation. This induction precedes the expression of the transcription factors, PPARγ and C/EBPα, and other adipocyte-specific markers including adipocyte fatty acid-binding protein. Although both kelch-like proteins and FRZB are known to affect the induction of C/EBPα and PPARγ, those genes were not differentially expressed between SD- and LD-housed cats in the present study. The lack of response may have been due to the food-restricted feeding protocol in our study. If fed *ad libitum*, it is likely that increased energy intake and adipose expansion would have occurred resulting in increased PPARγ and C/EBPα expression.

Keratins (KRT5, 14, 25, 74, and 85) and keratin-associated proteins (KRTAP8-1; KRTAP11-1), all related to cell development, were up-regulated in SD-housed cats (Table 2). Keratin filaments are abundant in keratinocytes in the cornified layer of the epidermis - cells that have undergone keratinization. Cytokeratins are expressed by mesenchymal stem cells of human adipose tissue [60]. Keratinocyte growth factor (KGF) is also expressed by mesenchymal stem cells in adipose tissue and functions in a paracrine fashion to stimulate epithelial cell proliferation and differentiation [61,62]. KGF mRNA is present in the stromal–vascular fraction of human adipose tissue [63], and its expression is up-regulated in early-life programmed rat model of increased visceral adiposity [64]. KGF is produced by rat mature adipocytes and preadipocytes as well as 3T3-L1 cells [65]. Importantly, Zhang et al. [65] demonstrated that KGF stimulates preadipocyte proliferation, but not differentiation, via an autocrine mechanism. Although the expression of several cytokeratins was influenced by photoperiod in this study, KGF expression was not altered. The role of keratin and keratin associated protein in adipose tissue is unclear and warrants further investigation.

The expression of chemokine (C-C motif) ligand 2 (CCL2) and CCL5, plasminogen activator inhibitor (PAI1), CD1e molecule, T-lymphocyte activation antigen CD80, intercellular adhesion molecule-1 (ICAM-1), T-cell immunoglobulin and mucin domain containing 4 (TIMD4), and TIM1 were lower in SD-vs. LD-housed cats (Table 3), suggesting reduced macrophage number and inflammation in adipose tissue. CCL2, also called monocyte chemoattractant protein 1 (MCP1), is believed to be an important initiator of adipose inflammation because it is important in attracting inflammatory cells into white adipose tissue. CCL2 expression is positively correlated with macrophage accumulation in adipose tissue, with overexpression being associated with obesity and insulin resistance [66-68]. CCL5 functions similarly by recruiting macrophages into adipose tissue and activating nuclear factor-kappa-B (NFκB) signaling [69,70]. PAI1 is a protease inhibitor that facilitates blood clotting. It is also thought to contribute to the development of insulin resistance and obesity, however, with its expression being induced by increased circulating free fatty acids [71-73]. Because adipose tissue macrophages express high levels of PAI1 [74], these data are in agreement with that of CCL2 and CCL5, and suggests increased macrophage infiltration in LD-housed cats in the current study. TIM1, TIMD4, and CD80, all expressed by macrophages, are involved in regulating T-cell proliferation and activation. ICAM-1 is a member of the immunoglobulin superfamily and is overexpressed during high-fat diet feeding or under conditions of inflammation [75,76]. Although common pro-inflammatory cytokines (e.g., TNFα; IFNγ; IL6) were not differentially expressed in our study, collectively, our data suggest a lower macrophage number or activity and a decreased level of inflammation in adipose tissue of SD-housed cats.

**Table 3:** Genes that had decreased expression in adipose tissue of SD- versus LD-housed cats

**Functional classification**	**Gene name**	**Gene symbol**	**Fold change**
Blood coagulation			
Blood coagulation	Plasminogen activator inhibitor 1	PAI1	-2.35
Cell development and structure			
Actin binding	fascin homolog 1, actin-bundling protein (Strongylocentrotus purpuratus)	FSCN1	-1.65
Cell growth and differentiation			
Cell differentiation	FOS-like antigen 1	FOSL1	-2.42
Cell growth and differentiation	growth differentiation factor 10	GDF10	-1.69
Immune function			
Immune function	chemokine (C-C motif) ligand 2	CCL2	-2.24
Immune function	C-C motif chemokine 5	CCL5	-2.84
Immune function	CD1e molecule	CD1E	-2.76
Immune function	T-lymphocyte activation antigen CD80	CD80	-2.78
Immune function	intercellular adhesion molecule 1	ICAM1	-1.58
Immune function	T-cell immunoglobulin and mucin domain containing 4	TIMD4	-1.72
Ion binding transport			
Ion transport	solute carrier family 22 (organic cation transporter), member 1	SLC22A1	-1.75
Metabolism			
CHO metabolism	aldolase C, fructose-bisphosphate	ALDOC	-1.69
Cholesterol metabolism	low density lipoprotein receptor	LDLR	-1.90
Cholesterol metabolism	oxidized low density lipoprotein (lectin-like) receptor 1	OLR1	-3.87
Cholesterol metabolism	apolipoprotein L domain containing 1	APOLD1	-1.96
Lipid metabolism	glucose-6-phosphate dehydrogenase	G6PD	-1.69
Lipid metabolism	adiponutrin	ADPN	-1.79
Lipid metabolism	stearoyl-CoA desaturase (delta-9-desaturase)	SCD	-1.96
Metabolism	RAS-like, family 10, member A	RASL10A	-1.86
Mitochondria metabolism	solute carrier family 25 (mitochondrial carrier; citrate transporter), member 1	SLC25A1	-1.67
Mitochondria metabolism	solute carrier family 36 (proton/amino acid symporter), member 2	SLC36A2	-1.70
Protein processing			
Protein processing	T-cell immunoglobulin and mucin domain containing 4	TIM1	-2.05
Protein processing	heat shock 105 kDa/110 kDa protein 1	HSPH1	-1.53
Transcription			
Transcription	regulatory factor X, 2 (influences HLA class II expression)	RFX2	-1.83

Inflammation and oxidative stress not only alter energy homeostasis and host metabolism, but contribute to insulin resistance as well. Fasting blood glucose concentrations were not different between groups in our study [14]. Blood insulin was not measured. Photoperiod has the potential to manipulate these processes because melatonin is a powerful free radical scavenger [77] and regulator of antioxidant enzymes [78]. Recently, melatonin has also been shown to modify inflammatory proteins such as TNFα and ICAM1 [79] and improve insulin sensitivity in obese rats [80]. Ankyrin 1 (ANK1), ANK3, ankyrin repeat and suppressor of cytokine signaling (SOCS) box containing 4 (ASB4), ASB14, and ASB15, were up-regulated in SD-housed cats (Table 2). ASB proteins are involved in cellular differentiation and appear to regulate components of the insulin signaling pathway. ASB4, specifically, is down-regulated in the hypothalamus of rats during fasting and with obesity [81,82]. ASB4 appears to influence insulin signaling by inhibiting c-Jun NH_2_-terminal kinase (JNK) activity [83], but further research is necessary to clarify its role in adipose tissue. Several genes associated with calcium transport, including ATPase, Ca++ transporting, plasma membrane 2 (ATP2B2), calcium channel, voltage-dependent, alpha 2/delta subunit 1 (CACNA2D1), phospholamban (PLN), solute carrier family 8 (sodium/calcium exchanger), member 3 (SLC8A3), and ryanodine receptor 3 (RYR3), were also up-regulated in SD-housed cats (Table 4). Intracellular Ca^2+^ plays an important role in adipocyte lipid metabolism and triglyceride storage, modulating energy storage in this tissue. Although these changes have been associated with improved insulin sensitivity, response to glucose or insulin dose was not tested in this study. Uncoupling proteins (UCP) are most widely known for their ability to separate oxidative phosphorylation from ATP synthesis, resulting in thermogenesis, but appear to have other functions as well. UCP3, expressed in muscle and adipose tissues, was up-regulated in SD-housed cats and has been shown to be up-regulated by PPARγ [84], increases mitochondrial Ca++ uptake [85], and is thought to limit the production of reactive oxygen species [86].

**Table 4:** Genes associated with blood coagulation, ion binding and transport, and metabolism that had increased expression in adipose tissue of SD- versus LD-housed cats

**Functional classification**	**Gene name**	**Gene symbol**	**Fold change**
Blood coagulation			
Blood coagulation	multimerin 1	MMRN1	2.16
Ion binding and transport			
Ion binding	phosphotriesterase related	PTER	3.57
Ion binding	zinc finger protein 385B	ZNF385B	3.45
Ion transport	ATP-binding cassette, sub-family C (CFTR/MRP), member 8	ABCC8	4.92
Ion transport	ATPase, Na+/K + transporting, beta 4 polypeptide	ATP1B4	4.11
Ion transport	ATPase, Ca++ transporting, plasma membrane 2	ATP2B2	3.22
Ion transport	calcium channel, voltage-dependent, alpha 2/delta subunit 1	CACNA2D1	2.65
Ion transport	cholinergic receptor, nicotinic, delta (muscle)	CHRND	7.49
Ion transport	phospholamban	PLN	2.81
Ion transport	solute carrier family 8 (sodium/calcium exchanger), member 3	SLC8A3	3.57
Ion transport	solute carrier family 9, subfamily A (NHE2, cation proton antiporter 2), member 2	SLC9A2	3.30
Ion transport	ryanodine receptor 3	RYR3	3.30
Metabolism			
AA metabolism	glutamate decarboxylase-like 1	GADL1	4.34
CHO metabolism	epilepsy, progressive myoclonus type 2A, Lafora disease (laforin)	EPM2A	2.41
Glycogen metabolism	protein phosphatase 1, regulatory subunit 3A	PPP1R3A	4.60
Glycogen metabolism	amylo-alpha-1, 6-glucosidase, 4-alpha-glucanotransferase	AGL	3.35
Lipid metabolism	phospholipase A2, group IVE	PLA2G4E	3.32
Lipid metabolism	protein kinase (cAMP-dependent, catalytic) inhibitor alpha	PKIA	2.33
Lipid metabolism	protein phosphatase 2, regulatory subunit B”, alpha	PPP2R3A	2.50
Metabolism	ADP-ribosyltransferase 3	ART3	3.41
Metabolism	isopentenyl-diphosphate delta isomerase 1	IDI1	2.21
Metabolism	ATP/GTP binding protein 1	AGTPBP1	1.71
Metabolism	uncoupling protein 3 (mitochondrial, proton carrier)	UCP3	4.10

In the current study, several genes associated with lipid and carbohydrate metabolism were altered due to photoperiod, but not with clear implications on feline health. Expression of stearoyl-CoA desaturase (SCD), adiponutrin (ADPN), and glucose-6-phosphate dehydrogenase (G6PD), low density lipoprotein receptor (LDLR), oxidized low density lipoprotein receptor 1 (OLR1), and apolipoprotein L domain containing 1 (APOLD1), all involved in lipid and cholesterol metabolism, were down-regulated in SD-housed cats (Table 3). Epilepsy, progressive myoclonus type 2A, Lafora disease (laforin) (EPM2A), protein phosphatase 1, regulatory subunit 3A, and amylo-alpha-1,6-glucosidase, 4-alpha-glucanotransferase (AGL), all involved in glycogen metabolism, were up-regulated in SD-housed cats (Table 4).

Glycogen storage is much lower in adipose tissue compared to skeletal muscle and liver, but is present and believed to yield precursors for glycerol formation and lipid deposition [87,88]. SCD is the rate-limiting step in the synthesis of unsaturated fatty acids [89], and appears to change with BW. Reduced adiposity of the SCD-knock out mouse has been attributed to reduced lipid biosynthesis and increased lipid oxidation [90], while increased SCD in obese subjects seems to favor lipid storage [91-93]. ADPN is highly expressed in adipose tissue and appears to play a role in lipogenesis, being positively correlated with lipogenic enzymes and negatively correlated with uncoupling protein-1 (UCP1) [94]. G6PDH is a metabolic cytosolic enzyme involved in the pentose phosphate pathway, which provides NADPH for tissues actively engaged in the biosynthesis of fatty acids and/or isoprenoids. G6PDH is highly expressed in adipocytes of obese animal models and is involved with adipocytokine expression, oxidative stress, and insulin resistance [95,96]. LDLR and OLR1 internalize LDL and oxidized LDL, respectively. LDL is the primary carrier of cholesterol in the bloodstream, with LDLR playing an important role in regulating blood cholesterol concentrations. OLR1 is one of a group of scavenger receptors, which are transmembrane proteins involved in several cellular functions, including adhesion and elimination of apoptotic cells and modified lipoproteins such as oxidized LDL. Oxidized LDL internalization leads to macrophage infiltration and secretion of proinflammatory cytokines [97]. OLR1 expression has been shown to be regulated by adiponectin and PPARγ [98,99], but also associated with insulin resistance [99]. Collectively, these gene expression differences suggest a difference in adipose tissue metabolism between SD- and LD-housed cats, with SD-housed cats having an increased level of glycogen synthesis, but lower level of lipogenesis in the fasted state.

Because this study has several limitations, data interpretation requires caution. One limitation was that our study only includes gene expression data. Because our adipose tissue biopsies were quite small, each sample was used entirely for RNA isolation and sequencing. Thus, we were not able to perform histological or any other analysis on those samples to compare adipocyte size, number, or responsiveness. Second, circulating melatonin was not measured in this study because a validated method for feline melatonin measurement is not currently available. Third, gene expression profiles were based on the entire adipose biopsy sample rather than a specific cell type. Lastly, cats were fed to maintain BW to limit changes to photoperiod. Therefore, the differences observed here may be different than cats allowed to adjust BW according to season that is known to occur in many seasonal animals. Despite the limitations provided above, this study provides a novel dataset of the adipose tissue transcriptome changes in response to photoperiod in healthy cats.

## Conclusions

Photoperiod is known to cause physiological changes in seasonal animals and has been shown to affect energy intake, energy expenditure, and voluntary physical activity levels in cats. The molecular mechanisms by which these changes occur and the possible role that adipose tissue plays, however, are unknown. Our mRNA abundance data suggests that short day housing promotes adipogenesis, minimizes inflammation and oxidative stress, and alters lipid and carbohydrate metabolism in adipose tissue of cats, even when fed to maintain BW. Future research is needed to confirm these results in cats fed *ad libitum* and allowed to adjust food intake and BW according to photoperiod. Research designed to link gene expression of specific adipose cell types to pertinent physiologic outcomes is also justified.

## Availability of supporting data

The dataset supporting the results of this article is available in the Gene Expression Omnibus repository at the National Center for Biotechnology Information (NCBI) archives (http://www.ncbi.nlm.nih.gov/geo) under accession #GSE46431 (GSM1130067-GSM1130086).

## Competing interests

The authors declare that they have no competing interests.

## Authors’ contributions

KSS designed the research experiment; KLK conducted the animal experiment and collected adipose tissue; AM performed laboratory analysis; and AM, ACD, and KSS wrote the paper. All authors read and approved the final manuscript.
